# Rapid Oral Health Deterioration in Older People—A Narrative Review from a Socio-Economic Perspective

**DOI:** 10.3390/jcm12062396

**Published:** 2023-03-20

**Authors:** Linda Slack-Smith, Gina Arena, Lydia See

**Affiliations:** 1 School of Population and Global Health M431, The University of Western Australia, 35 Stirling Highway, Crawley, WA 6009, Australia; 2 School of Dentistry, The University of Queensland, 288 Herston Road, Herston, QLD 4006, Australia; 3 School of Dentistry, The University of Western Australia, 35 Stirling Highway, Crawley, WA 6009, Australia

**Keywords:** oral disease, older adults, rapid oral health deterioration, social determinants of health

## Abstract

Poor oral health is a common morbidity in old age with older adults less likely to attend dental care and more likely to have dental disease; this situation is exacerbated by older adults retaining more teeth often with complex restorations. Evidence suggests that some older adults experience rapid oral health deterioration (ROHD). While more clinical and population level evidence is needed, current evidence suggests upstream changes addressing disadvantage through the social determinants of health (SDH) may impact broader disorders such as ROHD, often occurring as older adults become dependent. The aim of this paper is to conduct a narrative review to explore the social determinants of ROHD in older adults. The social determinants of health are important in understanding oral health including ROHD. This includes the important influence of the economic determinants. We explored the SDH as relevant to oral health and ROHD including using a framework based on that of the Fisher-Owens conceptual model (for children) but adapted for older adults. Better understanding of these relationships is likely to assist in future prevention and care.

## 1. Introduction

“No one should be denied access to life-saving or health promoting interventions for unfair reasons, including those with economic or social causes. These are some of the issues being addressed by the Commission on Social Determinants of Health. When health is concerned, equity really is a matter of life and death.” (the then Director-General World Health Organization, Dr Margaret Chan, 2007) [1].

Oral disease is one of the most common health conditions globally with a substantial burden across the life-course including older adults [2,3]. Oral disease in older adults comprises a number of conditions including dental caries, periodontitis, xerostomia and a range of other conditions [4]. Studies consistently show a decline in dental service attendance and poorer oral health as older adults age [2,5]. While the number and proportion of older adults is increasing globally, the decrease in edentulism along with substantial numbers of complex restorations means not only a high demand for geriatric dental care but also the need for more complex dental care in this cohort [6]. Beliefs are changing with less acceptance that tooth loss and poor oral health are a natural consequence of normal ageing [7,8]. Evidence suggests that poor oral hygiene and poor diet are strong predictors of dental caries and periodontal disease—both of which are often considered preventable [9]. In addition, periodontal disease (and tooth loss) has been associated with significant comorbidities such as cardiovascular disease, diabetes and respiratory disease [9]. We know from the current literature that some medications can influence oral health in older adults, probably through impacts on saliva [10].

Rapid oral health deterioration (ROHD) occurs when oral health declines more rapidly than expected [11]; the concept was developed to describe and deal with those older adults who have a substantial decline in health followed by a decline in oral health [11]. We also know that oral disease is related to our patterns of life and particularly social determinants of health (SDH) and that individuals who are disadvantaged and have a lower socio-economic status are disproportionally affected [12]. There is a dearth of longitudinal total population or large population level cohort data in older adults so it is difficult to follow trajectories of oral health in this age group as have been explored in other age groups [13]. It is also difficult to look at associations with SDH at the population level [14]. However, we do know consistently from the literature that older adults often reach a point where many health and social issues are impacted as they lose capacity to care for themselves [15]. This is often the point where people enter aged care or receive in-home care [16]. There are two potential scenarios for ROHD: one where an older adult faces general decline (aligning with the definition given above) and another where it is particularly a dental problem without obvious general decline. There are a dearth of data on such nuances of the problem and as noted above, the term has primarily been used to understand oral health decline associated with general health decline [11].

Oral health is influenced by factors across the life-course and while key factors such as sugar in diet and fluoride in water and toothpaste are of central importance, there are many social and medical factors that are likely to be important [3,17]. The social determinants of health (SDH) influence the health and oral health of a population across the life-course [18], and are important to consider in terms of oral health for older adults. The SDH include various social, economic and environmental factors that shape health outcomes, including education, employment, access to healthcare, income and the physical environment [18]. Social determinants of health that are reflected in smoking, high alcohol consumption and the inability to access good quality food and nutrition over time are also predictors of poor oral health in older adults [19]. Other factors associated with social determinants include costs of dental treatment and access to transport, social isolation and lack of role models for good oral health and hygiene [16]. It has previously been reported that oral morbidity often occurs earlier in life for individuals with less financial security [20]. However, these poor outcomes may be exacerbated in older adults with reduced mobility and cognitive function, along with financial barriers, poorer access to healthy nutrition and difficulties accessing transport which may impact dental services [12].

Older adults may become frail although this should not be assumed for all older adults. Hakeem and colleagues conducted a systematic review to investigate frailty and oral health. They concluded that there were strong longitudinal associations between oral health and frailty with oral health being a potential predictor of frailty and with nutrition as a potential mediator [21]. Similarly, Kimble and colleagues identified an association between the decline in muscle strength and oral health using data from two cohort studies [22]. Often at a point where things deteriorate, there are multiple co-morbidities and social issues to address, yet a lack of interdisciplinary primary care means this trend is often only noted at the stage when things start to go very wrong [23]. One outcome that may occur in an older person is ROHD. The literature is limited regarding ROHD in older adults, even more limited when it comes to exploring associated factors such as social determinants of health.

In addition to individual level pathology, it is also important to acknowledge the context where broader socioeconomic factors can also undermine oral health in older adults. This would include factors that can impact macro level changes such as health policies and other interagency collaboration and initiatives that have a direct impact on oral health by addressing root causes and having long-term outcomes. As recognized by Watt, it is at this level where we might need effective upstream prevention [24,25]. Understanding what matters to participants in relation to oral health as they age is also important [26]. Current evidence of what is supposedly preventable oral disease in older adults suggests that upstream factors such as a siloed approach to oral health care and a focus on treatment rather than prevention is clearly not adequate to meet the changing needs of the current ageing population [17]. This includes increasing numbers of older adults with full or partial dentition [16,18].

A recent systematic review on oral health in older adults found that an important component of socioeconomic status was the educational attainment of individuals [27]. Individuals with a higher level of education had better oral health prevention practices and more access to healthcare services. Being able to identify oral health conditions, monitoring and implementing oral health plans can help to reduce the burden of oral health disease in this population [27].

One of the ways we can consider factors associated with ROHD is via the Fisher-Owens’ conceptual model, developed to understand children’s oral health and the complex interplay of risk factors [28]. The model includes five key components related to the SDH: genetic and biological factors, the social environment, the physical environment, health behaviors and dental and medical care. These components influence oral health outcomes and contribute to the dialogue surrounding oral health at a population level and can be adapted to suit older adults by reflecting on oral health influences across the life-course. A key aspect of the SDH is that they do not act in isolation of each other but instead through multiple and complex interactions [29].

The Seattle Care Pathway offers important guidance re care in this age group [30] Yeung [19] suggests that oral health care systems require an inter-sectoral and interprofessional approach to adapt to oral health in older adults that includes dental professionals; policymakers; health, medical professionals and public health professionals; and researchers. Yeung also suggests a plan of action that integrates oral care into primary health care, promotes oral health across the life-course and informs evidence-based oral health policies. Given the target group is the ageing population, many of whom are retired, Yeung [19] also suggests removing financial and physical barriers to accessing oral care and engaging and training appropriate stakeholders to provide oral health care to maintain the oral health of older adults.

The aim of this paper is to explore the SDH as potentially associated with ROHD in older adults. This will comprise consideration of the concept and evidence for ROHD in the context of poor oral health in older adults, exploration of the potential influence of social determinants with a focus on economic factors and consideration of potential future approaches.

## 2. Methods

This is a narrative review focusing on peer-reviewed literature and using appropriate grey literature when required.

## 3. Results

We will consider the issue of poor oral health in older adults, the more specific issue of ROHD, potential associations with factors with a focus on the SDH and potential strategies for prevention and care with ROHD.

### 3.1. Poor Oral Health of Older Adults

Poor oral health in older adults is a neglected and intractable problem, impacting on overall health and wellbeing of the older adults. It can affect their quality of life in many ways, including limiting the pleasure of eating, because of reduced chewing capacity with resultant malnutrition, feeling ashamed of dentures, and association with diabetes and mental health [2,3,4]. It can increase hospital admissions related to poor dental care such as aspiration pneumonia and dental care under general anesthetic as well as the broader impact of dental problems including malnutrition and falls [31,32]. Additionally, there is an increase in polypharmacy and medications’ use with side effects of xerostomia resulting in an increased risk of oral diseases such as dental caries [33].

There is also the impact on dental treatment required. Barriers to optimal care include lack of specific training for dental professionals and aged care staff [19,20,21], overcoming patient fears related to past experiences of oral care [22], family attitudes to oral care [23] and lack of understanding of the importance of oral care and cost of and access to dental services [24,25].

### 3.2. The Concept of Rapid Oral Health Deterioration

Firstly, it is important to explore the concept of ROHD in older adults [11,34]. It is important both dental professions and other relevant professions and carers understand and can identify ROHD. There is limited but important work in the literature looking at measures of ROHD in older adults [11,35]. Meanwhile, the term “rapid deterioration of oral health” or “rapid oral health deterioration” has been used in various research articles—some of that work is not relevant here—such as when used in another age group or a particular context.

Prior to examining the factors associated with poor oral health, particularly ROHD, it is important to consider what is happening in older adults at a more systemic level. Whether oral health is impacted or not is associated with the individual’s ability to care for themselves and their mobility to access oral health care. The physiology of ageing can be characterized by the progressive loss of physiological integrity, resulting in impaired function and increased vulnerability [36]. All of these can result in the development of a set of unifying pathological conditions of geriatric syndrome [37,38]. This syndrome is a complex relationship between multimorbidity, polypharmacy and frailty that can contribute to poorer oral health outcomes [33]. It can be used as a framework for addressing the complex oral health needs of older adults and the presentation of common geriatric oral syndromes [33,39]. A complex array of shared risk factors (e.g., increased age, cognitive impairment, functional impairment and impaired mobility) can result in the traditional geriatric syndrome (incontinence, falls, pressure ulcers, delirium and functional decline) and may be influenced by multi-morbidities (e.g., diabetes, neurodegenerative disorders, Alzheimer’s/dementia and cardiovascular disease), polypharmacy and resultant frailty [33,39]. All of which have an impact on oral health with the presentation of a common set of symptoms (geriatric oral syndrome) such as burning mouth syndrome, xerostomia, dental caries, periodontitis, dysgeusia, dysphagia and dyskinesia/dystonia [33,39]. Ní Chróinín and colleagues found that poor oral health in older adults was associated with Alzheimer’s disease and kidney failure—even when adjusted for medication and salivary pH [6]. Therefore, in addressing the oral health of an older adult, it will mean developing a preventive and management strategy that takes into consideration the impacts of the presence of geriatric syndrome as well as in the broader context of SDH. So, in understanding ROHD, geriatric syndrome may be part of the pathway in which SDH can influence ROHD.

There are at least two ways we could explore this topic. Firstly, we could determine changes in oral health (with appropriate measure for dental caries, periodontitis, tooth loss, etc.) in older adults with longitudinal studies of older adults (potentially concurrent with regular dental appointments) with associated data on SDH. Unfortunately, there are limited cohort data on older adults and a dearth of dental data. Collecting such data may be costly (without good administrative systems) and require some time, however this would be valuable longer term, especially if population-based and linkable with broader administrative data to help identify SDH [14]. Thus, it is more feasible that we could consider likely pathways for ROHD in older adults by reviewing the existing literature on oral health determinants more broadly. Consideration of the social gradient and inequalities in oral health may help further determine the specific factors disadvantaging older adults to target preventative oral health measures including policies and practices.

Frailty is an important consideration in terms of risk for ROHD, although with varied definitions, and work on frailty and oral health is still very much in progress [40]. One group at particular risk of ROHD are older adults with dementia [41]. We know generally that people with dementia have poorer access to dental services and poorer oral health outcomes than other older adults [42].

It is important that both dental professions and other relevant professions understand and can identify ROHD as a precursor to understanding the influence of social determinants. There is limited but important work in the literature looking at measures of ROHD in older adults [11,35]. Marchini and colleagues have developed a teaching tool to establish risk of ROHD in older adults and this was found useful in teaching geriatric oral health for dental students over a number of years [11,35]. It would be useful if such tools were considered for wider use in dental teaching.

### 3.3. Factors Associated with Poor Oral Health and Rapid Oral Heath Deterioration

Influences on oral health in older adults, including ROHD, are complex and affected by a range of social, heath and clinical factors. We will focus here on the social determinants. If we consider the impact of SDH on oral health, their importance is evident, although substantial work remains before obtaining a full understanding of the relationships between SDH and oral health in older adults and a translation of the findings. The SDH are underpinned by social justice. There is a substantial social justice issue when our older adults are impacted in terms of pain, poor ability to eat properly with reduced pleasure in eating and subsequent effects on wellbeing (including social isolation due to reduced social interactions because of embarrassment, shame, reluctance to smile because of their oral health status) because they are not receiving adequate dental care, this being worst in the more marginalized groups [1]. There are a number of pathways through which disadvantage in terms of social determinants impacts on oral health outcomes. Dental attendance is often affected and our own work using population level data from a national survey, shows that lower dental attendance is strongly associated with older age, less schooling, lower wealth and higher measures of disadvantage [5].

While Fisher-Owens et al. developed a model regarding oral health in children (with influences from Bronfenbrenner’s work) some of the principles could be applied here [28,43]. Adapting Fisher-Owens, we could have the following categories of influences (see Table 1) [28].

These influences do not fully address what occurred at different points during the life-course but instead largely reflect the current situation in relation to older age and ROHD. There also may be several overlaps between the different influences such as health behaviors that are part of both older adult and family as these key influences are linked to the environment and characteristics.

### 3.4. Potential Strategies for Dealing with Rapid Deterioration of Oral Health

The problem of ROHD is complex and it is likely that multiple approaches will be required to deal with the problem. Watt has identified the importance of upstream action in oral health [25]. Successful prevention could potentially mean more people with intact teeth and limited restorations, but this may take generations. Broader upstream actions include addressing social determinants more broadly. Education has strong socio-economic impacts on oral health outcomes. This would likely also apply in terms of impacts on ROHD. In addition, developing better integrated primary care for older adults, ideally with inclusion of a dental professional but also with skills in non-dental professionals to identify and predict this disorder. This is important as many older adults do not see a dental professional regularly [5].

We have limited dental professionals with appropriate training in dental care for older adults; in fact, in many countries we still have limited training in geriatrics for dental students. Slack-Smith et al. have noted the limited emphasis on geriatric dentistry in dental courses in Australia and others similarly overseas [44]. The current model of dental care, which largely depends on ad hoc access to a limited supply of public and private dentists working in residential aged care sector is clearly not adequately addressing this problem in Australia.

Due to such complexity of the interplay of risk factors, comorbidities, polypharmacy and frailty, the complexity of oral health management for these individuals increases. The health outcomes of frailty come with increased risk of falls, disability, dependency and death [45]. Therefore, it will be important to identify any determinants that could indicate that the individual is at the precipice of decline so that oral health interventions can occur with adequate timing and prior to any occurrence of ROHD. This is particularly important in the case of older adults in high-dependency residential facilities as these individuals are at the greatest risk of rapid health decline and by extension oral health decline. It is this identification of the window of opportunity that is currently elusive to dentistry.

One way of identifying the time frame in which oral health assessment, prevention and intervention can occur is utilizing the tools that consider factors of SDH. These are factors that can be assessed by non-dental professionals. As such, they can then give a quantifiable indication in which these determinants can then trigger a referral to an oral health care professional for further oral health assessments that may be required in the context of an expected deterioration in health as well as the level of oral health care intervention that is most suitable for the expected trajectory of the individual. This modality of assessment and care intervention looks at oral health in terms of not a disease but as a form of holistic care that puts the patient at the center of care.

Additionally, the concept of the Geriatric 5Ms of: mind, mobility, medications, multi-complexity and matters most can be used as a framework in the oral health management plans of older adults [46]. These 5Ms are domains in which they allow the consideration of functional status, medication reviews, careful evaluation of risks and benefits of treatment, assessment of goals of care as well as prognosis of the patient’s condition to be incorporated into the overall management and comprehensive treatment planning of the older adult [46]. They consider the patient in a holistic manner and put the patient’s beliefs and values at the center of care. Thus, the 5Ms allow management to be patient centered and not disease centered.

## 4. Discussion

This paper has focused on consideration of the issue of ROHD and the potential role of various factors focusing on SDH, including economic determinants. While clinical and public health evidence in this area is very limited, our growing knowledge of SDH and oral health is valuable in considering likely factors and pathways. There is no simple way of looking at ROHD or fixing it, it is a complex and multilayered problem [47]. However, we will probably never have adequate individual care systems to provide adequate care to resolve the problem of ROHD at a population level. If using a population health framework, it may help turn the perspective from treating oral disease to preventing oral disease, in this case ROHD. Fisher-Owens’ adapted model provides this framework for understanding the complex interplay between various social determinants and the impact on ROHD for older adults. We argue that we need quality clinical knowledge and an understanding of associated factors and an improved understanding of causal pathways. MacEntee noted the need for better models in this area, noting many current health promotion models are not adequate for looking at the oral health of older adults [48]. This model offers a tool for healthcare professionals, policymakers and researchers to address health disparities among older adults that are not solely determined by medical interventions but also shaped by broader societal and environmental factors.

The consideration of ensuring the latest research data can be translated into action at a policy and clinical level will allow for the effective and efficient address of oral diseases. There needs to be a process in which evidence-based data can be readily and rapidly applied to clinical practice. It may mean simplifying bureaucratic processes to allow for more efficient and effective policy changes for such translatability to occur. Over the last 30 years, there has been an observed rise (as seen in the WHO’s recent global oral health status report 2022) in estimated case numbers of oral diseases, growing by more than 1 billion [49]. There was a 50% increase in oral disease case-numbers, which is higher than the population increase of about 45% during the same period [49]. What is more concerning is the rate of increase in case numbers of oral diseases is higher than the demographic growth across countries of varying degrees of socio-economic status [49]. Therefore, it can be argued that the increasing prevalence of oral diseases is a cause for concern and may even indicate a silent crisis that could result in increased public expenditure in dealing with the individual and systemic repercussions of untreated oral diseases. The COVID-19 pandemic demonstrates at a global scale, in times of crisis, the ability to rapidly and efficiently expedite regulatory processes to allow for the development of vaccines and medications which would otherwise have taken years to occur. In this context can this not be applied to addressing the state of global oral health? This is particularly crucial in the context of ROHD in the elderly. ROHD, if left untreated, can have a downstream effect of poor health, and quality of life and in extreme cases, systemic infections resulting in increased hospitalizations and potential death from infection. Therefore, it becomes imperative that research should be undertaken in such a way that new data are readily translatable with concurrent influences on public policy such that it allows for the rapid dissemination of information and applications into clinical practice.

The strengths of the study include a novel approach and the application of the Fisher-Owens’ model in older adults. There are limitations with a narrative review but it is an appropriate step for understanding the issue. A narrative review was provided as a first step in the exploration of this area as it helped us to identify gaps in the literature and highlight the areas requiring further investigation. As we develop our understanding and body of evidence, a systematic review would then be useful to help provide a more systematic and rigorous search process. This information can help to inform the development of search strategies, potential biases or limitations in the existing evidence and assist with a design that addresses these issues.

ROHD is likely to be a more extreme manifestation of normal dental disease. We need to better understand the clinical characteristics while exploring associated determinants in terms of the SDH. An adapted Fisher-Owens’ model offers a potential framework to explore influences. Reducing serious poor health outcomes in older adults and supporting those who do experience RODH is important. Looking at our systems in terms of structures such as interdisciplinary care can be very powerful. We need to broadly disseminate information about this disorder not only to dental professionals, but all professionals and families engaged in elder adult care and consider most effective research questions and approaches to improve outcomes.

## Figures and Tables

**Table 1:** Influences on oral health in older adults-adapted from Fisher-Owens [28].

**(Individual) Older adult influences:** These influences would include the biology and genetics, the current state of dental care, behaviors and practices, the role of dental insurance and other financial support influencing oral health.
**Family, care and care facility influences:** The ongoing relation to socio-economic status, ability to access dental care, other interdisciplinary care, communication, decision making, safety, family function, culture, social practices and social support.
**Community level influences:** The type of dental services (systems), social environment, dental care system characteristics, social capital, physical environment and community oral health environment.

## Data Availability

Data sharing not applicable to this article as no datasets were generated or analyzed during the current study.
